# Tubulointerstitial nephritis and uveitis syndrome in an adolescent female: a case report

**DOI:** 10.1186/s13256-021-03017-8

**Published:** 2021-09-04

**Authors:** Tadej Petek, Maja Frelih, Nataša Marčun Varda

**Affiliations:** 1 Department of Pediatrics, University Medical Center Maribor, Ljubljanska ulica 5, Maribor, Slovenia; 2 Institute of Pathology, Faculty of Medicine, University of Ljubljana, Korytkova 2, Ljubljana, Slovenia; 3 Faculty of Medicine, University of Maribor, Taborska ulica 8, Maribor, Slovenia

**Keywords:** Tubulointerstitial nephritis with uveitis, Acute tubular injury, Bilateral uveitis, Renal biopsy

## Abstract

**Background:**

Tubulointerstitial nephritis with uveitis syndrome is a rare disease affecting mainly children and young women. Tubulointerstitial nephritis with uveitis is a diagnosis of exclusion, requiring a high degree of clinical suspicion. Studies report recent infections or certain drugs as precipitating factors of a lymphocytic oculorenal immune response. The prognosis is usually favorable with topical and systemic corticosteroid therapy.

**Case presentation:**

We report a literature review and the case of a 14-year-old white girl, who presented to the ophthalmology department with features of one-sided uveitis. Upon transfer of patient to nephrological care, diagnostic work-up revealed renal involvement. Renal biopsy showed a mixed-cell and granulomatous tubulointerstitial nephritis with some noncaseating granulomas, leading to a diagnosis of tubulointerstitial nephritis with uveitis syndrome. With topical ocular and systemic corticosteroid therapy, the patients’ condition improved over several weeks.

**Conclusions:**

Our case highlights the importance of early recognition and treatment of this syndrome, where cross-specialty care typically leads to a favorable outcome.

## Background

Tubulointerstitial nephritis and uveitis (TINU) syndrome is a rare multisystem autoimmune disorder, presenting mainly with oculorenal pathology. Since its first recognition by Dobrin *et al*. [1], more than 300 cases have been reported worldwide [2]. Here we present the case of a 14-year-old girl who presented to our department with unilateral anterior uveitis and concomitant signs of acute interstitial nephritis. Upon extensive work-up, the diagnosis of TINU syndrome was confirmed.

The case is worth to be presented as it showed elevated erythrocyte sedimentation rate with only mild urine and biochemical abnormalities, but important histological changes on renal biopsy. Also, a review of published case series is provided.

## Case presentation

A 14-year-old white girl with unilateral anterior uveitis and abnormal urinalysis was referred from the ophthalmological care to our nephrology unit in November 2018. She had been seen at our out-patient clinic at the age of 12 years in 2016. Her family history was positive for elevated blood pressure. She was the first child of an uneventful pregnancy, born at 40 weeks gestation with a birth weight of 3990 g and birth length of 53 cm.

Upon visit, she was seen due to elevated blood pressure with occasional tension-type headaches, obesity, impaired glucose tolerance, and hyperlipidemia, which were a result of a sedentary lifestyle and unhealthy eating habits. She reported no first-degree family members with metabolic syndrome or its complications. Ambulatory blood pressure monitor values were within reference ranges (average 24-hour systolic and diastolic blood pressure 117 and 66 mmHg, respectively), so we implemented nonpharmacological lifestyle approaches. During follow-up, she received extensive evaluation, including endocrinological and dietary assessment, and was continued to be seen by our pediatric nephrologist twice per year.

In October 2018, aged 14 years, she presented to the Department of Ophthalmology with 1 week of redness, pain, epiphora, and loss of visual acuity of the right eye. She denied any recent drug exposure, allergy, infection, or symptoms of systemic illness. A diagnosis of acute anterior uveitis was made, followed by topical corticosteroid and cycloplegic treatment, which led to symptom alleviation.

### Investigations

**Fig. 1:**
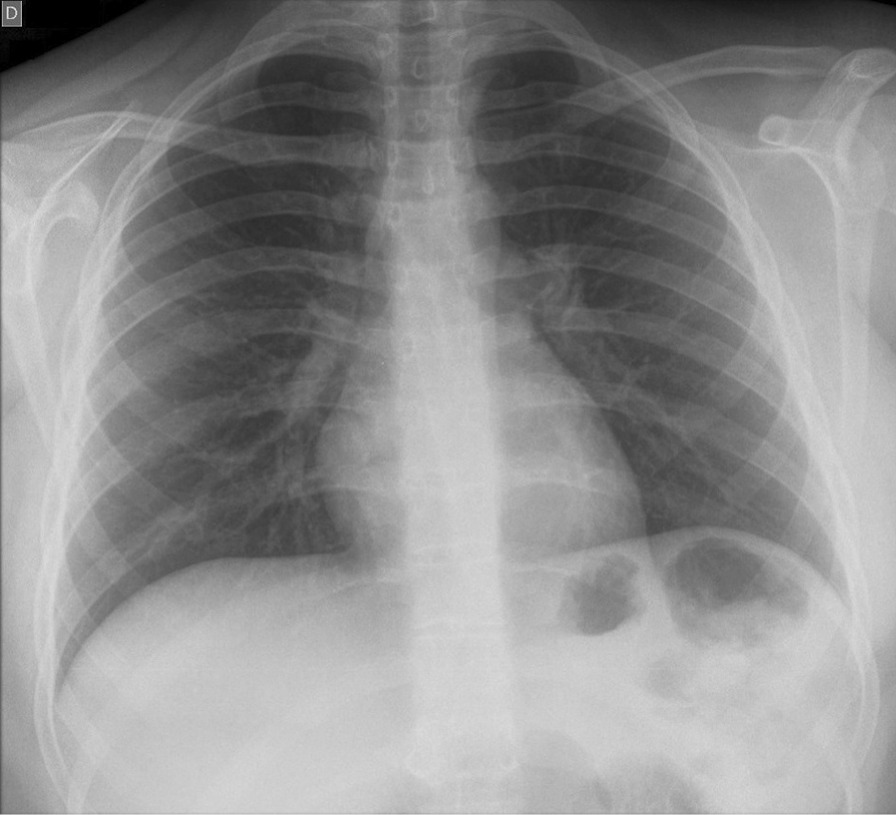
Chest radiograph in posteroanterior view. The radiological appearance of lung parenchyma was normal, which aided in ruling out sarcoidosis, a possible differential diagnosis

A broad diagnostic work-up was performed. Renal ultrasound was normal. Also, chest radiography was also normal (Fig. 1), which in conjunction with a normal serum angiotensin-converting enzyme level and absence of cough excluded sarcoidosis, a known oculorenal offender. However, upon laboratory evaluation, marked elevation in erythrocyte sedimentation rate (ESR, 98 mm/hour), mild elevation of serum C-reactive protein (CRP, 13 mg/L), mild normocytic anemia (Hb, 113 g/L), elevated serum creatinine (80 µmol/L), mild proteinuria (0.38 g/day), microalbuminuria (urine albumin-to-creatinine ratio, 58 mg/g), elevated values of alpha-1 microglobulin (urine alpha-1-microglobulin-to-creatinine ratio, 3.24 mg/g), and normoglycemic glycosuria (1+) were observed. Immunological screening revealed elevated C3 complement fraction (C3, 2.01 g/L), with negative antinuclear antibodies (ANA), anti-extractable nuclear antigen antibodies (ENA), anti-deoxyribonucleic acid antibodies (anti-DNA) and antineutrophil cytoplasmic antibodies (ANCA) antibodies. These values indicated mild renal involvement and prompted a referral to our nephrology unit.

Upon admission, she had no history of unexplained fevers, weight-loss, or other systemic symptoms. She had a pulse of 100 beats/minute, blood pressure of 126/81 mmHg, and body temperature of 36.5 °C. Her review of systems was negative, with a gradual improvement of symptoms and vision of the right eye. She continued both-sided topical cycloplegic and topical corticosteroid therapy. Borderline blood pressure values with repeated and persistent abnormal values of ESR, serum urea and creatinine, proteinuria, and glucosuria, indicating kidney injury, prompted a kidney biopsy.

**Fig. 2:**
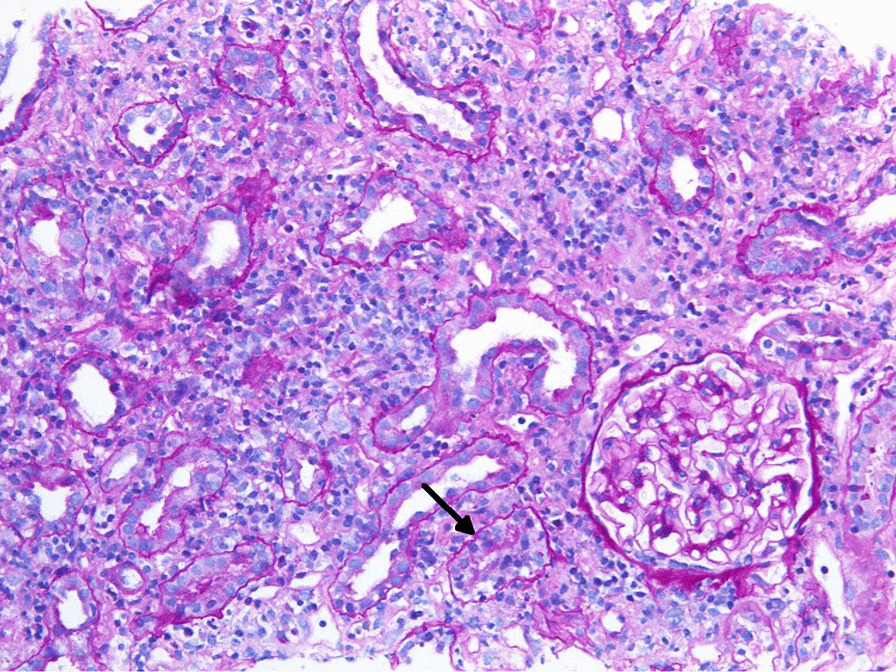
Renal biopsy sample A. Tubulointerstitial nephritis with acute tubular injury and a focus of mononuclear tubulitis (arrow). Interstitial infiltrate is composed of mononuclear cells (lymphocytes and macrophages with fewer plasma cells). Glomeruli were unremarkable [Periodic acid–Schiff (PAS), 200×]

**Fig. 3:**
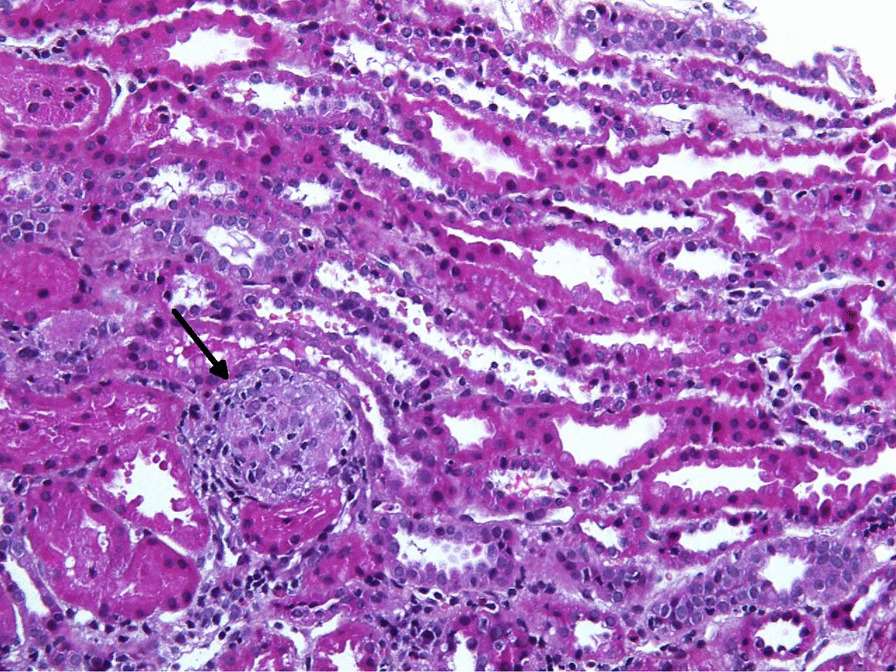
Renal biopsy sample B. Noncaseating granuloma (arrow) in the interstitium (Modified Hematoxylin-Eosin (MHE), 200×)

Histopathology revealed focal tubulointerstitial nephritis. Interstitial inflammatory cell infiltrate was composed of lymphocytes, macrophages, fewer neutrophils, eosinophils, and plasma cells and rare noncaseating granulomata, with foci of invasion of lymphocytes into the tubules (tubulitis). Tubules in the affected areas showed signs of acute tubular injury—flattened, irregular, and vacuolated tubular epithelium. Glomeruli and vessels were unremarkable (Figs. 2, 3). Immunofluorescence was negative. Electron microscopy showed no specific pathological findings. On the day of renal biopsy, 1 month after first symptom presentation, she also developed contralateral, left-sided anterior uveitis. A diagnosis of TINU syndrome was confirmed, based upon histopathological findings.

DNA typing of HLA loci showed the subtype HLA-B *07, *51; DRB1 *11, *13; DQA1 *05:05/05:09; DQB1 *03:01, negative for uveitis-related HLA-B27 genotype. Next-generation sequencing did not demonstrate any disease-related variants.

### Treatment

The patient was started on methylprednisolone 60 mg daily, which improved the laboratory markers of kidney injury and allowed us to continue an alternate-day corticosteroid therapy regimen. She also received pantoprazole 40 mg daily, trimetoprim–sulfametoxazole 480 mg twice daily every other day for *Pneumocystis carinii* pneumonia prevention, and vitamin D supplementation 2000 units daily, together with topical ocular therapy (scopolamine, nepafenac, dexamethasone). Because of elevated blood-pressure readings, she began therapy with ramipril 2.5 mg and later 5 mg daily and received regular follow-up.

### Outcome and follow-up

**Fig. 4:**
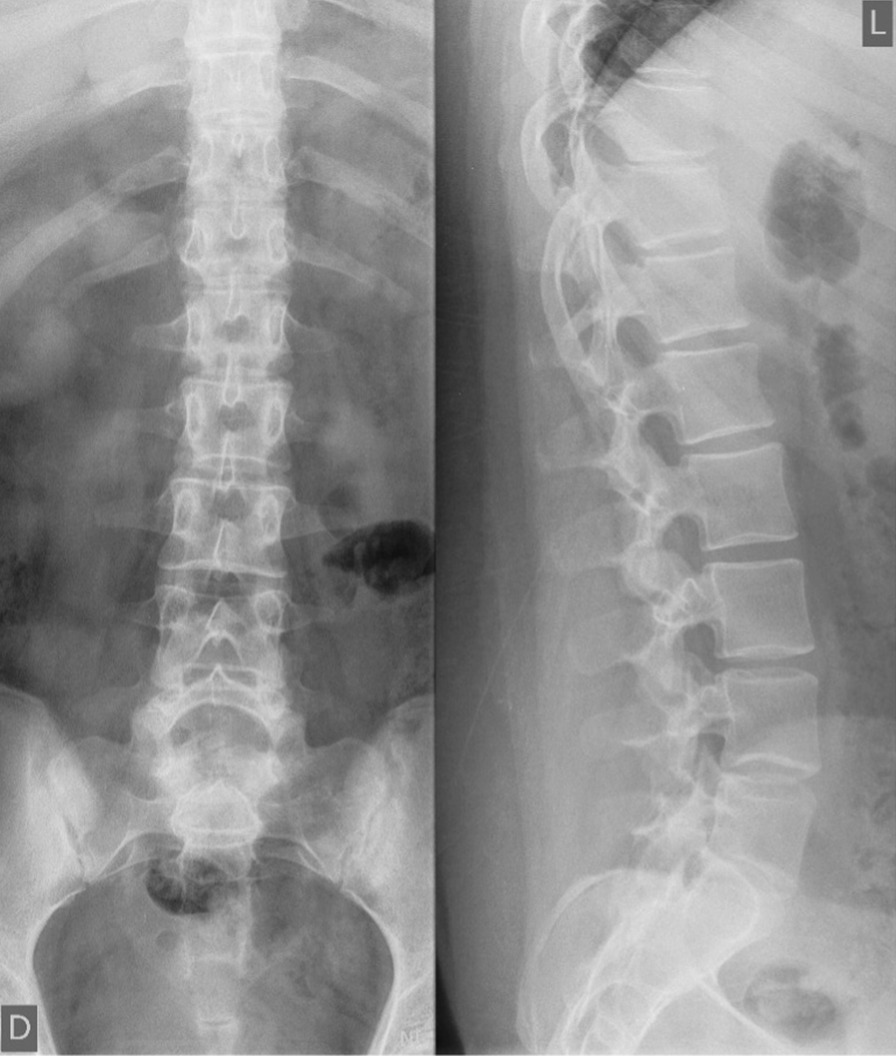
Lumbosacral radiograph—anteroposterior and lateral views. The radiograph of the lumbosacral spine did not show any fractures, which might occur with the patients’ prolonged corticosteroid therapy

After 3 months, upon evaluation at our out-patient clinic, her ocular symptoms improved, although she started having pain in her lumbar spine. Clinical examination showed a Cushingoid appearance with a 4 kg increase in body weight since discharge. Blood pressure values with antihypertensive therapy were normal. Lumbosacral spine X-ray imaging was normal, without signs of osteopenia (Fig. 4). This allowed a slow reduction in corticosteroid therapy upon following weeks and motivated her for implementation of healthy lifestyle measures.

At the most recent ambulatory office visit, two and a half years after onset of TINU, the patient denied any further ocular exacerbations, but she gained weight and had a body mass index of almost 35 kg/m^2^. Her 24-hour ambulatory blood pressure values were normal, as well as renal ultrasound examination, without presence of renal scarring. She was receiving ramipril 2.5 mg and metformin 500 mg twice daily each, together with education on necessary lifestyle changes.

## Discussion and conclusions

**Fig. 5:**
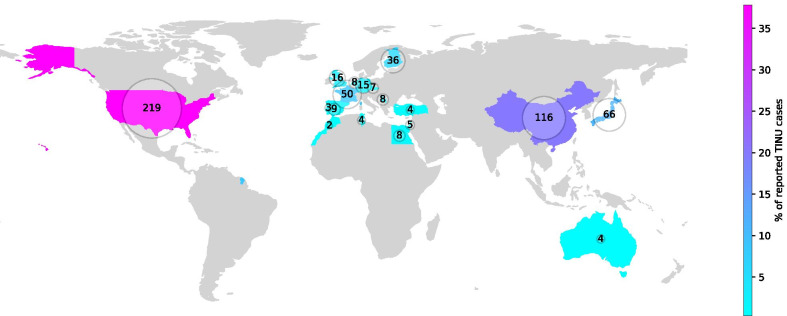
World map of published case series of TINU syndrome. Countries with published case series of TINU syndrome are presented, with a sum of 580 cases worldwide

Our article presents, to the best of our knowledge, the first Slovene case of TINU syndrome in a 14-year-old girl in published literature. The diagnosis was suspected by the presence of renal and ocular findings, combining acute interstitial nephritis and anterior uveitis, and confirmed by renal biopsy. Current literature suggests that approximately 300 cases have been published [2]. We present a literature review of 580 described cases. Table 1 presents the case series published in the last 10 years. Countries with published case series are presented in Fig. 5 and in greater detail in Table 2.

**Table 1:** List of published case series with two or more patients, published after the year 2010

	No. of patients	Presentation	Laboratory findings	Renal biopsy	Treatment	Outcome	Important contribution
Weinstein *et al*. (2010), Israel [34]	5	Four women and one man aged 18-64 years with systemic symptoms, all with renal (and two with ocular) involvement at initial presentation	Moderate-to-severe renal dysfunction, proteinuria, elevated ESR, anemia	Renal biopsy in all patients, with moderate-to-severe interstitial nephritis	All Pt on systemic steroids, three on either cyclosporin or azathioprine	Uveitis relapse in 3/5 Pt after corticosteroid cessation, necessitating immune-modulatory agents	Adult patients with TINU had more severe uveitis than previously reported
Biester *et al*. (2010), Germany [35]	2	13-year-old boy and 5 years later 17-year-old girl with acute anterior uveitis	Pt 1: systemic inflammation, renal dysfunction Pt 2: elevated serum β-2 microglobulin, proteinuria	Pt 1: interstitial nephritis Pt 2: no signs of interstitial nephritis on repeated renal biopsies	Pt 1: topical and systemic steroid; mycophenolate-mofetil after frequent relapses Pt 2: topical prednisolone	Frequent relapses of acute uveitis in 1/2 Pt	Two familial TINU cases with specific HLA-DQB1 and -DRB1 alleles
Tan *et al*. (2011), People’s Republic of China [4]	9	Seven female and two male Pt; mean age 45.2 years	Acute kidney injury (mean serum creatinine 241 µmol/L), mCRP serum autoantibodies in all Pt	Renal biopsy in all TINU cases; all samples positive for mCRP immunohistochemistry in tubules and interstitium	Topical ocular steroid in all patients, oral prednisone in 8/9 patients (median 30 mg/day), cyclophosphamide in 2/9 patients	Serum creatinine of all Pt normalized within 2–4 months after therapy initiation	High prevalence of serum anti-mCRP autoantibodies in patients with TINU syndrome
Houghton *et al*. (2012), Oregon [36]	4	Three male and one female Pt aged 13–36 years	NA	Lymphocyte-dominant interstitial inflammation accompanied by lymphocytic tubulitis	NA	NA	TINU is usually not associated with IgG4 sclerosing disease
Birnbaum *et al*. (2012), Illinois [37]	2	5-year-old and 51-year-old patient. Rash in 1/2 Pt	Both Pt with elevated serum β-2 microglobulin	NA	NA	None had active chronic disease	Simultaneous-onset bilateral acute anterior uveitis is more common in younger patients and in TINU
Peräsaari *et al*. (2013), Finland [18]	20	Ten male and ten female Pt, median age 12.8 years. Anterior uveitis in 20/20 Pt. Two Pt had uveitis prior to nephritis, 11 simultaneous with and 7 Pt ≥1 month after nephritis onset.	NA	Biopsy-proven AIN (not otherwise specified)	NA	All PT followed-up by a pediatric ophtalmologist and monthly by a pediatric nephrologist for at least 12 months	Strong associations exist between certain HLA genotypes in TIN(U) patients
Saarela *et al*. (2013), Finland [2]	16	Eight male and eight female PT. Median age of uveitis onset 12 years and 9 months. Bilateral uveitis in all Pt. No ocular symptoms in 8/16 Pt	NA	Biopsy-proven AIN (not otherwise specified)	All 16 Pt received topical steroids. Mydriatics and antiglaucoma therapy used in 9/16 and 6/10 Pt, respectively. Prednisone or placebo per trial protocol	Follow-up duration between 6 and 48 months	No statistically significant difference in occurrence of uveitis in AIN patients, treated with prednisone or placebo
Takemoto *et al*. (2013), Japan [38]	2	12-year-old girl and a 12-year-old boy with probable TINU syndrome	Important elevation of urinary β-2 microglobulin in both patients	Not performed.	Pt 1: after several ocular inflammatory exacerbations with systemic steroid therapy, intravitreal bevacizumab proved useful Pt 2: topical and systemic corticosteroid	Pt 1: without choroidal neovascularization in 5 years’ time Pt 2: NA	Two cases of choroidal neovascularization in TINU, one successfully treated with intravitreal bevacizumab injection
Li *et al*. (2014), People’s Republic of China [39]	31	Mean age 47 years, with a 5.2:1 female predominance. Median time from onset of symptoms to renal biopsy was 30 days	Increased serum creatinine, increased urinary α-1 microglobulin excretion and decreased urine osmolality in all Pt. Approximately 50% of Pt had elevated urinary NAG excretion and leukocyturia	Performed in all patients, together with mCRP-antibody and Krebs von den Lungen-6 assays	Systemic prednisone for 6–8 weeks and subsequently tapered. 10/31 Pt received methylprednisolone pulse therapy. Cyclophosphamide used in 11/31 Pt	Median follow-up period 37 months. Approximately one-third of patients had relapses during follow-up, and most had incomplete renal recovery	Uveitis in TINU can present well after onset of AIN, leading to misdiagnosis. Elevated mCRP-antibody levels may be useful to predict late-onset uveitis occurrence
Reddy *et al*. (2014), Virginia [21]	6	Four boys and two girls with definite TINU, median age of 11 years. Diagnosis of renal disease before uveitis by a median of 3 months	NA	NA	All Pt received oral corticosteroids. 3/6 Pt treated with methotrexate and 4 Pt with mycophenolate mofetil, and one each received infliximab or cyclosporine	Median follow-up was 3.5 years. 2/6 Pt who completed therapy were successfully weaned from immunosuppressive therapy.	Panuveitis is underappreciated as a manifestation of TINU
Ali *et al*. (2014), Oregon [40]	4	One Pt with definite and three with “possible or probable” TINU diagnosis, aged 10–31 years	Pt 1: mild anemia, elevated ESR and CRP, normal urinalysis Pt 2: elevated serum creatinine Pt 3: elevated serum creatinine and urine leukocyte esterase Pt 4: normal lab values	1/4 Pt with biopsy-proven interstitial nephritis	Pt 1: topical and systemic steroid Pt 2: systemic steroid and oral methotrexate Pt 3: oral steroid, switched to methotrexate Pt 4: topical steroid, mydriatic, subsequently oral steroid	Regular follow-up for up to 3 years	Chorioretinal lesions should be recognized as a component of TINU
Hettinga *et al*. (2015), Netherlands [41]	8	Two definite and six probable TINU cases, aged 12–20 years.	All PT had increased serum creatinine values, 7/8 Pt had increased urinary β-2 microglobulin levels	2/8 with biopsy-proven AIN	NA	NA	Urinary β-2 microglobulin and serum creatinine are a simple diagnostic screening tool for detecting renal dysfunction in TINU
Legendre *et al*. (2016), France [42]	41	25 females and 16 males with biopsy-proven TINU. Median age at disease onset 46.8 years. 29/41 Pt had a bilateral anterior uveitis, and 24/41 presented with deterioration in general health	Moderate proteinuria in 32/41 Pt, sterile leukocyturia in 25/36 Pt. Median estimated GFR was 27 ml/minute per 1.73 m^2^	All Pt had AIN, 19/39 with light-to-moderate fibrosis and 5 Pt with acute tubular necrosis	36/41 Pt treated with oral corticosteroids, median duration of 8.0 months.	After 1 year of follow-up, 32% of patients suffered from moderate-to-severe chronic kidney disease, and 40% of Pt had uveitis relapses	Use of oral corticosteroids in TINU was associated with fewer uveitis relapses, but not better kidney function
Sobolewska *et al*. (2016), Germany [28]	9	Five female and four male Pt mean age 16.7 years. All presented with bilateral uveitis	Elevated urinary β-2 microglobulin levels in 8/9 Pt	3 Pt with biopsy-proven AIN. In 2 pediatric cases, parents declined renal biopsy	Mean follow-up of 19.6 months. 1 Pt with recurrences after 133 months of treatment	Mean follow-up period was 54.8 years	TINU syndrome characterized by limited responsiveness to corticosteroid therapy and less by severe complications
Sawai *et al*. (2016), Japan [43]	2	Two 14-year-old girls. Pt 1 had systemic symptoms and low back pain 4 days after third dose of HPV vaccination. Pt 2 had anterior uveitis 10 weeks after third dose of HPV vaccine	Pt 1: elevated CRP, serum creatinine, leukocyturia, glycosuria, and proteinuria Pt 2: elevated serum creatinine, glycosuria, proteinuria, hematuria	Renal biopsy-proven AIN in 1/2 Pt	Topical and systemic steroid in both cases	Pt 1 has stable renal function and long-term topical steroid therapy for uveitis. Pt 2 without symptoms after steroid therapy cessation	HPV vaccine might be causally related to TINU syndrome
Ariba *et al*. (2017), Tunisia [44]	4	Two male and two female patients aged 41–70 years. 1/4 Pt had fever, 3/4 Pt weight loss	Acute renal injury in 4/4 Pt. ESR elevated in all Pt, CRP in 3/4 Pt. ANCA positive 1:80 in 1/4 Pt	Renal biopsy performed in 1 Pt, consistent with AIN, without interstitial fibrosis	All patients initially received topical steroids. Systemic steroid started at onset of renal symptoms with tapering over 5-month period.	Renal outcome favorable in all Pt	The presentation and recognition of TINU in adult patients is probably underestimated
Nagashima *et al*. (2017), Japan [45]	3	Pt 1: 15-year-old boy with bilateral anterior uveitis Pt 2: 14-year-old girl with bilateral papilledema Pt 3: 49-year-old woman with panuveitis	Pt 1: elevated IgG, elevated ESR and CRP, azotemia, elevated urinary β2 microglobulin and NAG Pt 2: normal at initial visit Pt 3: mild increase in serum creatinine	Pt 1: biopsy-proven AIN Pt 2: no biopsy approach Pt 3: biopsy-proven AIN 12 months before admission	Pt 1: pulse of methylprednisolone 1 g/day for 3 days, tapering of dose Pt 2: topical steroid, triamcinolone acetonide Pt 3: topical and systemic steroid	Pt 1: continued topical and oral steroids needed due to relapse Pt 2: no relapse Pt 3: no relapse	In addition to anterior uveitis, TINU may present also with fundal features
Jia *et al*. (2018), People’s Republic of China [22]	38	NA	NA	All Pt with clinicopathologically diagnosed AIN	NA	NA	Patients with drug-induced AIN or TINU have genetic susceptibility in HLA-DQA1, -DQB1, and DRB1 alleles
Provencher *et al*. (2018), Iowa [46]	9	9 TINU Pt with iridocyclitis and elevated urinary β-2-microglobulin, 9/9 met full diagnostic criteria	Mean urinary β-2 microglobulin at presentation was 6536 μg/L (40.9 times the upper limit of normal); elevated serum creatinine in 7/9 Pt; proteinuria in 5/9 Pt	Performed in 3/9 Pt. All biopsies showed acute TIN	All Pt were treated with topical steroids, and oral steroids were used in 8/9 Pt. Two Pt were also treated with mycophenolate mofetil	Mean follow-up was 36.2 months. Relapse occurred once in two different Pt. An exacerbation occurred in 7/9 Pt within the first year	Urinary β-2 microglobulin correlates with uveitis activity and trends down over the course of TINU
Kanno *et al*. (2018), Japan [47]	5	Two male and three female Pt; mean age of 15.8 years. First presentation to ophthalmology in 4/5 Pt, pediatrics 1/5 Pt	Serum creatinine slightly increased in 2/5 Pt. Proteinuria in 3/5 Pt, glycosuria in 4/5 Pt, elevated urinary β-2-microglobulin in all Pt	2/5 Pt underwent renal biopsy, showing focal AIN	All Pt received topical steroids, 3/5 needed also systemic steroid because of renal manifestations	Mean follow-up of 54.0 months. Two Pt had recurrence of nephritis after steroid tapering. One Pt developed ocular hypertension on steroid therapy. Recurrence-free periods ranged from 12 to 71 months	Urinary β2 microglobulin level and HLA typing (especially HLA-DR4 or DRB1) may help in the diagnosis of TINU
Zhang *et al*. (2018), People’s Republic of China [48]	4	Age 10.8–13.6 years	All Pt presented with proteinuria and elevated urinary α-1-microglobulin. The ratio of urinary α1-microglobulin to microalbumin was greater than 1	NA	NA	All Pt seen within a 3-year period	A ratio of urinary α1-microglobulin to microalbumin greater than 1 can be used as a diagnostic criterion for tubuloproteinuria
Pereira *et al*. (2018), Portugal [49]	3	Pt 1: 13-year-old female presenting with bilateral anterior uveitis Pt 2: 12-year-old female presenting with bilateral and intermediate uveitis Pt 3: 12-year-old female presenting with systemic symptoms	Pt 1: hypertension, raised inflammatory markers, decreased GFR, hypokalemia, metabolic acidosis, leukocyturia, glucosuria, hematuria, non-nephrotic proteinuria, and raised urine β2-microglobulin levels Pt 2: iron-deficiency anemia, elevated ESR, decreased GFR, leukocyturia, glucosuria, hematuria, and non-nephrotic proteinuria Pt 3: decreased GFR, leukocyturia, glucosuria, hematuria, non-nephrotic proteinuria, and raised urine β-2-microglobulin levels	Pt 1: diffuse mononuclear cell interstitial infiltrates, consistent with AIN Pt 2: no biopsy Pt 3: lymphoplasmacytic interstitial infiltrates consistent with AIN	Pt 1: ocular dexamethasone and mydriatics, oral prednisolone, amlodipine, and potassium citrate. Afterwards methotrexate Pt 2: mydriatics, topical corticosteroids, and oral deflazacort Pt 3: ocular and systemic corticosteroids, mydriatics, and methotrexate	Pt 1: 5 years follow-up Pt 2: at 18 months receiving methotrexate, on remission Pt 3: one episode of recurrent uveitis within 5 months observation period	Patients with uveitis need to be screened for renal disease
Yang *et al*. (2019), People’s Republic of China [30]	32	Female-to-male ratio was 1.46, mean age of onset 41.1 years. 20/32 Pt had uveitis after AIN. Fatigue was most common systemic symptom (30/32 Pt) and polyuria most common renal symptom (20/32 Pt)	2/32 Pt had anemia. Other laboratory data are NA	Diagnoses of AIN were all confirmed by renal biopsy	Topical and systemic steroids in all Pt, from 2 to 38 months. Immunomodulatory agents administered to 18/32 Pt	Mean duration of follow-up was 3.16 years. 50% of recurrences occurred during the first year	Ultra-wide-field fluorescence is sensitive in detecting the activity of uveitis and might be useful in monitoring disease progression
Takeuchi *et al*. (2019), Japan [50]	8	Eight TINU Pt within a cohort of 156 Pt with noninfectious uveitis	NA	NA	Topical steroid monotherapy in 6/8 Pt, other 2 Pt received long-term steroids	NA	Betamethasone eye drops, topical triamcinolone acetonide, and long-term, systemic corticosteroids were the major therapeutic strategies used for uveitis relapse or exacerbation
Abd *et al*. (2020), Egypt [51]	8	Eight TINU Pt within a cohort of 781 Pt with intermediate uveitis	NA	NA	NA	NA	40% of patients with intermediate uveitis had identifiable a systemic disease
Clave *et al*. (2019), France [25]	7	Five male and two female Pt, aged 10.1–14.5 years, all with bilateral uveitis and renal involvement	All Pt with elevated serum creatinine and lower GFR, other laboratory data NA	NA	Full-dose steroid treatment was maintained for 1 month in all Pt, followed by gradual tapering. Steroid treatment was continued for 6.0 months	All Pt showed a gradual improvement of renal function	Children with idiopathic AIN and prompt treatment have a better prognosis, and chronic kidney disease occurrence justifies long-term follow-up
Çakan *et al*. (2019), Turkey [52]	4	Three male and one female Pt, median age at diagnosis of uveitis 13.4 years. Bilateral anterior uveitis in 3/4 Pt. All had systemic manifestations	All Pt had renal manifestations as microscopic hematuria, glycosuria, and mild proteinuria	NA	All Pt received topical steroids, and 1 Pt needed systemic steroids and methotrexate.	NA	A simple urine test may help in establishing the diagnosis of TINU syndrome in uveitis patients
Cao *et al*. (2020), Ohio [53]	10	Six female and four male Pt with TINU and posterior ocular segment inflammation. Age distribution was bimodal (10–46 years and 77–83 years)	Mean urinary β-2-microglobulin levels were more than tenfold upper limit of normal. Serum creatinine was elevated in 6/10 Pt. Urinalysis was abnormal in 9/10 Pt	2/10 Pt underwent a renal biopsy, one of which was positive for moderate-to-severe acute AIN, consistent with TINU	11 of 20 eyes were initially treated with topical steroids only, and 2 Pt received oral steroids alone due to posterior segment involvement	Visual acuity was stable or improved for all but one patient who had a subretinal macular scar	Posterior segment inflammation in the setting of TINU is not uncommon
Roy *et al*. (2020), United Kingdom [54]	6	6 Pt from a cohort of 10 Pt with AIN, age range 6–16 years, male-to-female ratio 1:9	Lowest GFR in TINU Pt ranged from 7 to 30 ml/min/1.73 m^2^	All Pt had biopsy-proven AIN	All Pt received systemic steroid therapy	Latest follow-up was from 2 to 70 months. None had experienced a recurrence of AIN	There is a high proportion of TINU in a UK case series of biopsy-proven AIN in children
Kitano *et al*. (2020), Japan [55]	4	4 Pt with TINU from a cohort of 98 Japanese uveitis Pt	NA	NA	48% of the 98 Pt received only topical steroids, whereas 39/98 Pt received some form of systemic antiinflammatory therapy	80.9% of the eyes maintained a visual acuity of 20/20 at the final visit	Hypotony, serous retinal detachment, and pupil disorders can lead to visual loss in uveitis patients, including TINU
Total	306						

*NA* data not available, *No.* number, *Pt* patient, *ESR* erythrocyte sedimentation rate, *HLA* human leukocyte antigen, *NAG* *N*-acetyl-β-(d)-glucosaminidase, *mCRP* modified C-reactive protein, *AIN* acute interstitial nephritis, *GFR* glomerular filtration rate, *HPV* human papilloma virus, *ANCA* antineutrophil cytoplasmic antibodies, *UK* United Kingdom

**Table 2:** Reported case series of tubulointerstitial nephritis with uveitis, sorted by country and year of publication

Country (city)	Author and year of publication (ref.)	No. of patients
France		
NA	Azar 2000 [56]	2
Dijon	Legendre 2016 [42]	41
Marseille	Clavé 2019 [25]	7
USA		
California	Mandeville 2001 [3]	133
California	Levinson 2003 [19]	18
Oregon	Mackensen 2007 [57]	33
Oregon	Houghton 2012 [36]	4
Illinois	Birnbaum 2012 [37]	2
Virginia	Reddy 2014 [21]	6
Oregon	Ali 2014 [40]	4
Iowa	Provencher 2018 [46]	9
Ohio	Cao 2020 [53]	10
Serbia		
NA	Nikolić 2001 [58]	8
Spain		
Madrid	Gorrono-Echebarria 2001 [17]	3
Sevilla	Sanchez Burson 2002 [59]	6
Japan		
Hirosaki	Tanaka 2001 [60]	2
Hirosaki	Suzuki 2004 [33]	2
Okayama	Matsuo 2002 [61]	4
Nagasaki	Deguchi 2003 [62]	2
Hokkaido	Goda 2005 [63]	12
Sapporo	Kase 2006 [5]	17
Tokyo	Yanagihara 2009 [32]	3
Sapporo	Takemoto 2013 [38]	2
Kanazawa	Sawai 2016 [43]	2
Yokohama	Nagashima 2017 [45]	3
Gifu	Kanno 2018 [47]	5
Saitama	Takeuchi 2019 [50]	8
Tokyo	Kitano 2020 [55]	4
Germany		
Essen	Hudde 2002 [64]	4
Tübingen	Biester 2010 [35]	2
Tübingen	Sobolewska 2016 [28]	9
United Kingdom		
London	Baker 2004 [29]	6
Southampton	Howarth 2004 [65]	2
Scotland	Joss 2007 [66]	2
Liverpool	Roy 2020 [54]	6
Australia		
Melbourne	Lim 2005 [67]	2
Adelaide	Li 2008 [68]	2
Czech Republic		
NA	Svozilkova 2006 [69]	5
Prague	Dusek 2008 [70]	2
Morocco		
Casablanca	Mortajil 2006 [71]	2
People’s Republic of China		
NA	Yao 2007 [72]	2
Beijing	Tan 2011 [4]	9
Beijing	Li 2014 [39]	31
Beijing	Jia 2018 [22]	38
Beijing	Zhang 2018 [48]	4
Beijing	Yang 2019 [30]	32
Israel		
Beer Sheva	Weinstein 2010 [34]	5
Finnland		
Helsinki	Peräsaari 2013 [18]	20
Oulu	Saarela 2013 [2]	16
Netherlands		
Utrecht	Hettinga 2015 [41]	8
Tunisia		
Tunis	Ariba 2017 [44]	4
Portugal		
Lisbon	Pereira 2018 [49]	3
Egypt		
Alexandria	Abd 2020 [51]	8
Turkey		
Istanbul	Çakan 2019 [52]	4
		580 in total

*NA* data not available

### Pathophysiology

In 2001, Mandeville *et al.* [3] proposed diagnostic criteria for TINU syndrome, comprising clinical and histopathologic features. Since then, studies have tried to elucidate the underlying mechanism of disease. Limited data suggest that modified C-reactive protein (mCRP), a uveal and renal tubular autoantigen, might play a role in eliciting an IgG-mediated oculorenal immune response [4]. A novel human glycoprotein, Krebs von den Lungen-6, was also observed to be significantly increased in sera and distal renal tubules of TINU patients [5]. Furthermore, certain interleukin-10 polymorphisms have been found to be more prevalent in TIN/TINU patients, broadening our understanding of the genetic basis of the disease [6].

### Differential diagnosis and epidemiology

TINU syndrome was shown to represent 15–65% of cases of acute interstitial nephritis (AIN) in pediatric renal care centers [7, 8]. It is essential to distinguish it from other causes of AIN, either drug-induced, autoimmune, metabolic, malignant, or consequential to a variety of infectious causes [9–11].

A large case series [3] suggests TINU shows a 3:1 female-to-male predominance, with a median age of 15 years. A UK-based study estimated the incidence as 1 per 10 million population per year [12]. Several recent reviews confirmed peak incidence in adolescence and a female-to-male predominance [9, 13–16]. Genetic studies indicate a strong association with certain HLA haplotypes, especially variants DQA1, DQB1, DRB1, and DR14 [17–21]. No disease-associated HLA variants were confirmed in our patient.

### Clinical presentation

TINU patients typically present with a viral-like illness, after which renal dysfunction is discovered. Alternatively, in about 20% of cases (including this case), the patient presents with symptoms of burning eyes and/or visual blurring [9, 13–16] and is subsequently discovered to have renal manifestations. This asynchrony prompts a high degree of clinical suspicion in treating young, female patients with AIN or acute uveitis. In a Finnish study [2], which prospectively evaluated the presence of acute uveitis in biopsy-proven AIN at onset, at 3 and at 6 months afterwards, 16/19 (84%) of pediatric patients had uveitis within the observation period, half (8/16) without ocular symptoms. However, there are no guidelines or recommendations regarding ocular screening in patients with AIN.

Acute kidney injury is nearly universal in the setting of TINU and is usually in the mild-to-moderate range. It may be complicated with hypertension, which was also the case in our patient. In literature, cases of Fanconi syndrome and nephrogenic diabetes insipidus in association with TINU have been described [23, 24], as well as progression to chronic kidney disease in adult and pediatric patients [25]. Table 3 presents TINU characteristics in comparison with disease manifestations, seen in our patient.

**Table 3:** Comparison of clinical characteristics of tubulointerstitial nephritis and uveitis with our patient

Clinical characteristics^†^	Our patient
AIN in TINU	
1. Abnormal renal function	Mild elevation of serum creatinine. AH treated with lifestyle interventions already before disease onset
2. Abnormal urinalysis	
Low-grade proteinuria, glycosuria, urinary eosinophils, hematuria, sterile pyuria, and presence of white cell casts, as well as phosphaturia and aminoaciduria. Elevated urinary NAG, α-1 and β-2 microglobulin	Mild proteinuria, microalbuminuria, elevated α-1 microglobulin, normoglycemic glycosuria
3. Systemic illness lasting ≥ 2 weeks	No history of systemic symptoms
(a) Signs and symptoms: fever, rash, weight loss, anorexia, malaise, fatigue, flank pain, arthralgia or myalgia	Marked elevation of ESR, mild elevation of CRP, mild anemia
(b) Blood and urinary findings: anemia, eosinophilia, elevated ESR and CRP, abnormal LFT, acid–base disorders	
Uveitis in TINU	
1. Classical bilateral, anterior uveitis with ocular redness, pain, and photophobia	Unilateral anterior uveitis at presentation and contralateral anterior uveitis after 1 month
2. Atypical uveitis: intermediate and/or posterior involvement	
3. Complications: posterior synechiae, cystoid macular edema, disc edema, elevated intraocular pressure, cataract formation	

^†^The renal and ocular course are thought to be independent, and neither the severity nor prognosis of nephritis is influenced by the presence of uveitis [16]. *AIN* acute interstitial nephritis, *AH* arterial hypertension, *NAG* *N*-acetyl-β-d-glucosaminidase, *ESR* erythrocyte sedimentation rate, *CRP* C-reactive protein, *LFT* liver function tests. Adopted from [3, 9].

### Pathohistological findings

Upon light microscopy, features of a predominantly CD3-positive lymphocytic infiltrate with fewer plasma cells and macrophages are present. A prominent eosinophilic infiltrate may be seen initially, as well as interstitial granulomas that can become confluent. Upon disease progression, the inflammation subdues, while variable amounts of interstitial fibrosis appear. The CD4-to-CD8 ratio varies. However, studies indicate a reciprocal T-cell profile in the kidney as compared with what is seen in peripheral blood, indicating that cellular immunity is active at the tissue level and decreased systemically [9]. Tubular atrophy or tubulitis is also characteristic for TINU and is in accordance with clinical evidence of tubular dysfunction, reported in our patient and many cases of TINU [9, 26].

### Treatment

As in other uncommon disorders, there are no evidence-based treatment protocols, so the decision whether to initiate systemic corticosteroid or immunosuppressive therapy depends on renal and ocular involvement. If nephritis is mild or in remission, topical steroids may be used to treat uveitis, though not efficient in posterior intraocular segment involvement [9]. Systemic corticosteroids are generally reserved for cases of progressive renal involvement [3] and are needed in about 80% of patients [27]. Oral prednisone or prednisolone with a dosage of 1–1.5 mg/kg/day is usually used. The duration and schedule for tapering of steroid dose depends mainly on patient response [9, 13, 15, 16, 27]. Because of frequent relapses and recurrences of disease, some authors suggest at least 12 months of oral corticosteroid therapy [28], while others advocate an early and short course [29], which was the case in our patient. We believe that, in the absence of evidence-based treatment protocols, a case-by-case management may be adopted [27].

In a pediatric case series [2], all 16 patients with TINU received topical ocular steroids. Mydriatic therapy was necessitated in 9/16 patients and antiglaucoma therapy in 6/16 cases. Surprisingly, oral prednisone did not influence occurrence of uveitis. In steroid-resistant cases or in exacerbation of disease after weaning from corticosteroid therapy, immunosuppressive medications such as cyclophosphamide, cyclosporine, methotrexate, or mycophenolate mofetil may be used [9, 13, 15, 16].

### Prognosis

Ocular and renal outcomes are usually good with appropriate treatment, as most respond to initial topical or systemic therapy. The disease may remit altogether or run a chronic or recurrent course, usually appropriately controlled through judicious use of immunosuppressive agents [9, 20, 30]. Though early TINU literature held that renal disease often resolved spontaneously, repeat renal biopsy studies reported cases of continued nephritis after pulse corticosteroids [31, 32], mandating close follow-up of patients for several years after disease onset. Prompt corticosteroid therapy initiation also seems to play a role as demonstrated in a small case series, where a patient with delayed treatment demonstrated persistent elevations of beta-2-microglobulin and renal inflammation with subsequent renal damage [33]. Additionally, severe TINU can lead to end-stage renal failure requiring dialysis and kidney transplant [16].

Most patients will maintain or improve eyesight from presentation. However, ocular disease recurs in up to 50% of patients after corticosteroid withdrawal [16]. Younger age was identified as a risk factor for chronic uveitis, though few studies have evaluated the impact of systemic therapy on reducing that risk [16]. Vision is seldom severely impaired, as demonstrated in a case series of 133 patients [3], where vision outcome was rarely worse that 20/40. Therefore, optimal care incorporates joint nephrological and ophthalmic input, which was done in our patient.

## Conclusion

This case highlights the need to maintain a high degree of suspicion and close follow-up in young, female patients who present with features of tubulointerstitial nephritis or uveitis. As there are no evidence-based protocols for treating TINU, management relies on case reports and case-series. The recommended treatment for uveitis is topical steroids. However, most cases necessitate systemic therapy with corticosteroids owing to renal involvement or in cases of posterior ocular involvement.

Immunomodulatory drugs may be used in resistant cases. With prompt therapy, prognosis of both renal and ocular involvement is usually favorable, though relapses might occur. Therefore, combined nephrological and ophthalmological care is warranted. Furthermore, there is a need for a multicenter study and registry formation to obtain important clinical data regarding follow-up and treatment of these patients, and a frameshift for implementation of multinational guidelines of treatment and prognosis.

### Learning points

•In patients presenting with uveitis and/or acute interstitial nephritis, a suspicion of TINU syndrome should be made, especially if young and/or female.
•Even when histological features of important tubulointerstitial nephritis and noncaseating granulomata can be found, as in our case, the urinalysis can show only mild urine changes with proteinuria and glucosuria, with no hematuria. In our patient with bilateral uveitis and marked elevation of ESR, a renal biopsy proved useful in guiding therapy.
•With prompt ocular and systemic corticosteroid therapy, prognosis of TINU is favorable, despite occasional relapses.

### Patient’s perspective

I have to say that the overall care from both the Nephrology and Ophthalmology department was very professional. At first, I was quite shocked after I developed an inflammation of my eye, which did not allow me to see properly. Also, I was surprised that I had additional problems with my kidneys that I did not even notice. After several weeks of staying in the hospital, my nephrologist told me that I would have to undergo a kidney biopsy, the thought of which was quite scary. However, as I had already known the department staff for years, I trusted them fully and was not too worried after the diagnosis of TINU came. The therapy which was offered to me was quite tolerable, though I did not like gaining even more weight after being put on corticosteroids, which the doctors told me could happen. Coming home after more than a month, I am very happy to again attend school, and try to maintain a healthy lifestyle as recommended.

## Data Availability

Not applicable.

## Abbreviations

TINU: Tubulointerstitial nephritis with uveitis
ESR: Erythrocyte sedimentation rate
CRP: C-reactive protein
ANA: Antinuclear antibodies
anti-ENA: Anti-extractable nuclear antigen antibodies
anti-DNA: Anti-deoxyribonucleic acid antibodies
ANCA: Antineutrophil cytoplasmic antibodies
HLA: Human leukocyte antigen
mCRP: Modified C-reactive protein
AIN: Acute interstitial nephritis
